# Understanding the secondary outcomes of international travel measures during the covid-19 pandemic: a scoping review of social impact evidence

**DOI:** 10.1186/s12992-024-01064-6

**Published:** 2024-08-01

**Authors:** Kelley Lee, Salta Zhumatova, Catherine Z. Worsnop, Ying Liu Bazak

**Affiliations:** 1 Faculty of Health Sciences, Simon Fraser University, Blusson Hall, 8888 University Drive, Burnaby, BC V5A 1S6 Canada; 2 Pandemics and Borders Project, Faculty of Health Sciences, Simon Fraser University, Blusson Hall 8888 University Drive, Burnaby, BC V5A 1S6 Canada; 3 School of Public Policy, University of Maryland, College Park, MD 20742 USA

**Keywords:** Scoping review, COVID-19 pandemic, Travel measures, Secondary outcomes, Social impacts, Pandemic preparedness and response, International health regulations, Non-pharmaceutical interventions

## Abstract

**Background:**

Assessment of the effective use of international travel measures during the COVID-19 pandemic has focused on public health goals, namely limiting virus introduction and onward transmission. However, risk-based approaches includes the weighing of public health goals against potential social, economic and other secondary impacts. Advancing risk-based approaches thus requires fuller understanding of available evidence on such impacts.

**Methods:**

We conducted a scoping review of existing studies of the social impacts of international travel measures during the COVID-19 pandemic. Applying a standardized typology of travel measures, and five categories of social impact, we searched 9 databases across multiple disciplines spanning public health and the social sciences. We identified 26 studies for inclusion and reviewed their scope, methods, type of travel measure, and social impacts analysed.

**Results:**

The studies cover a diverse range of national settings with a strong focus on high-income countries. A broad range of populations are studied, hindered in their outbound or inbound travel. Most studies focus on 2020 when travel restrictions were widely introduced, but limited attention is given to the broader effects of their prolonged use. Studies primarily used qualitative or mixed methods, with adaptations to comply with public health measures. Most studies focused on travel restrictions, as one type of travel measure, often combined with domestic public health measures, making it difficult to determine their specific social impacts. All five categories of social impacts were observed although there was a strong emphasis on negative social impacts including family separation, decreased work opportunities, reduced quality of life, and inability to meet cultural needs. A small number of countries identified positive social impacts such as restored work-life balance and an increase in perceptions of safety and security.

**Conclusions:**

While international travel measures were among the most controversial interventions applied during the COVID-19 pandemic, given their prolonged use and widespread impacts on individuals and populations, there remains limited study of their secondary impacts. If risk-based approaches are to be advanced, involving informed choices between public health and other policy goals, there is a need to better understand such impacts, including their differential impacts across diverse populations and settings.

## Introduction

When the novel severe, acute respiratory syndrome coronavirus (SARS-CoV-2) was first reported to the World Health Organization (WHO) on 1 January 2020, declared a Public Health Emergency of International Concern (PHEIC) on 30 January 2020, and then characterized as a global pandemic on 11 March 2020, governments worldwide implemented a range of public health measures to control introductions and onward transmission of the virus (and later variants of concern) by travellers. International travel measures included advisories and warnings, screening (e.g. self-assessment protocols, temperature checks, testing), quarantine and/or isolation, immunity certification, entry and exit restrictions (e.g. suspension of non-essential travel), cancellation of transport services (e.g., flight bans, shutdown of cruise ships), and border (points of entry) closures. Virtually all countries applied some type(s) of international travel measure during the coronavirus disease (COVID-19) pandemic, although the types, timing, duration, stringency and targeting of such measures varied substantially across jurisdictions.

The widespread, wide-ranging and prolonged use of international travel measures during the pandemic proved unprecedented, prompting substantial study of their effectiveness in achieving public health goals, namely reducing virus introduction into new jurisdictions and onward transmission. A systematic review of 62 studies by Burns et al. found reduced introductions of a time-limited nature from the use of travel restrictions, screening and quarantine [8]. A review of 29 studies by Grépin et al. similarly found that early use of travel measures domestically in Wuhan, China, and internationally by selected countries, reduced the export of infections into new jurisdictions [20]. In 2023, after three years of travel measure use worldwide, a narrative synthesis of six systematic reviews found varying effectiveness of border closures/travel restrictions, symptomatic and diagnostic-based screening, and quarantine on travel-related onward domestic transmission of SARS-CoV-2 [19].

Despite this growing body of research, reviews to date conclude that “[t]he role of international border control measures in reducing transmission during the COVID-19 pandemic remains unclear and evaluating the effectiveness of such measures is challenging” [19]. This is due to limited confidence in the quality and reliability of evidence derived, in large part, from modelling (rather than observational) studies where “results depended on the assumptions that they made, not on real-life data” [8]. These studies apply different assumptions to analyze highly varied populations and national contexts. Importantly, studies also lack standardized terminology and definition to describe travel measures (as independent variable), and take limited account of the stringency of implementation (e.g., mandatory or voluntary, universal or targeted population, enforced or self-regulated). This lack of reliable, standardized and comparable data across jurisdictions on what and how countries used travel measures, is further challenged by data limitations on human mobility dynamics during the pandemic, and the incidence and prevalence of SARS-CoV-2 infection among travellers. These data challenges are unlikely to be resolved. Finally, studies vary in taking account of the coincidental use of non-travel-related public health measures, both internationally and domestically, as confounding factors.

Alongside these limitations to the evidence on the public health effectiveness of travel measures, there are substantial knowledge gaps about their secondary outcomes. Alongside better understanding of how effectively travel measures mitigated public health risks during COVID-19, many studies identify the need to take account of secondary, often unintended, consequences [61]. For example, restricting immigration during the pandemic, especially from low- and middle-income countries (LMICs) and countries undergoing conflict, on the grounds of public health protection, raised human rights concerns [41]. Restrictions on population mobility inflicted profound economic impacts on individuals, households, firms, sectors and economies [3, 48]. While Grépin et al. found existing “reviews included secondary outcomes, those outcomes were not considered in…[the authors’] knowledge synthesis” [19].

There are several reasons why fuller understanding of secondary outcomes of international travel measures is needed. First, policy makers seeking to advance public health goals may be undermining those goals by ignoring broader impacts of travel measures. For example, the adoption of the Temporary Restriction of Travelers Crossing the US-Canada Border for Non-Essential Purposes” on 21 March 2020 severely impacted travel by family members to care for ailing elderly relatives [11]. The exemptions adopted on 8 October, which included direct family members and travel on compassionate grounds, recognized the hardship caused by the restrictions [12, 50]. Second, given the central importance of equity in public health outcomes, measures that lead to disproportionate costs for some populations can undermine overall population health and well-being. For example, given inequitable access to COVID-19 vaccines and tests worldwide, the requirement by many countries to provide documentation prompted a booming market in counterfeit certificates [21]. This, in turn, undermined the overall ability to mitigate travel-related risk. Third, identifying which populations are impacted by secondary outcomes, especially those inequitably or severely affected, can inform adjustments to, or compensations for, travel measures. Restrictions on the transport of seafarers between ships (as their workplaces) and their countries of residence during the pandemic resulted in widespread hardship. Many seafarers were stranded at sea for many months beyond the normal contract period, while others were unable to be transported to ships to earn income. [27]. Measures to mitigate these impacts on seafarers, which support 80% of international trade flows, might include priority access to testing and vaccination, dedicated quarantine facilities, and benefits to replace lost income. Finally, measures that cause substantial and widespread negative social, economic and other secondary impacts can lead to a decline in public support, and even compliance, with pandemic response efforts. This, in turn, can have wider implications for longer-term public trust in government and public health institutions [4].

The improved weighing of secondary outcomes against public health costs and benefits is thus supported as a core component of the shift towards so-called “risk-based approaches” to travel measure use. The “[c]onsiderations for implementing a risk-based approach to international travel” set out by WHO [63], for instance, include economic impact, human rights, and the health and well-being of “vulnerable travellers, such as refugees, migrants and temporary or seasonal workers whose livelihoods largely depend on cross-border activities.” At the High Level Conference on COVID-19 held by the International Air Traffic Authority (IATA) in November 2021, member states committed to a “multilayer risk management strategy for international civil aviation, which is adaptable, proportionate, non-discriminatory and guided by scientific evidence in close cooperation and coordination with public health sector, with agreed practices harmonized to the greatest extent possible and underpinned by regular review, monitoring and timely information sharing among States” [26]. Towards addressing the knowledge gap on the secondary outcomes of travel measures, and to advance risk-based approaches, this paper conducts the first scoping review of existing evidence on their social impacts during COVID-19. The authors review the existing evidence on the economic impacts of travel measures as secondary outcomes in a separate scoping review [3]. The aim of this review is to assess the scope, methods, travel measures assessed, and social impacts identified in the existing literature. The findings inform recommendations for strengthening the evidence base on the social impacts of travel measures as secondary outcomes, and as a core component for advancing risk-based approaches to travel measure use in future PHEICs.

## Background

There is now substantial evidence of wide-ranging social impacts arising directly from the COVID-19 pandemic, and indirectly from the public health measures taken in response by governments worldwide [2, 39, 58, 64]. This evidence shows that “pandemics are as much about their social and economic implications as they are about their medical and health ones” [6]. Studies of the social impacts arising from COVID-19 countermeasures have tended to group international and domestic travel measures together, focused on restrictions to population mobility, and to group travel measures with other interventions (e.g., lockdowns). Conversely, while systematic reviews of the public health effectiveness of travel measures report “concerns about the unintended harms of those policies” [5], we are aware of only one evidence review of the unintended consequences arising from travel measures [31].

This review advances understanding of the social impacts of international travel measures in two ways. First, we apply a standardized taxonomy of travel measures to understand their social impacts. The varied application of travel measures during COVID-19 resulted in different, inconsistent, and sometimes even inaccurate terminology in research, policy and practice on their use. This is impeding empirical efforts to assess both the public health effectiveness, and secondary outcomes of travel measures during the pandemic. We define an *international travel measure* as a policy or intervention applied for the purpose of managing human mobility between two or more countries. We then developed a typology of cross-border travel and trade measures based on six categories: policy goal, type of movement, level of jurisdiction applied, public or private sector, stage of journey, and degree of restrictiveness [34]. This enabled us to develop a taxonomy of COVID-19 travel measures (summarized in Table 1) to code the WHO Public Health and Social Measures dataset, a repository of interventions adopted by WHO member states in response to the pandemic [66]. These standardized terms and definitions provide a clear starting point for identifying which travel measures were associated with which social impacts in the studies reviewed.

**Table 1:** Taxonomy of travel measures adopted during the COVID-19 pandemic

Travel measure type	Definition
Travel advisory or warning	Health advice or warnings provided by government on country-level transmission of COVID-19 to guide individual decisions on travel. This may include information on how to avoid virus (e.g. wear masks)
Health screening	Evaluation of the health or exposure status of a traveller entering a country (we separate out testing and providing additional travel documents as travel measures). This includes temperature checks, travel history, and monitoring of symptoms at points of entry (self-assessed or otherwise)
Quarantine	Requirement that an international traveller be separate from other people in a designated location (e.g. home, hotel, government facility), for a designed period of time, if they have or may have been exposed to SARS-CoV-2 infection to prevent onward transmission and monitor for illness
Testing	Requirement that an international traveller undergoes, or provides evidence of, a valid COVID-19 test before, enroute, during, and/or after their arrival at their destination
Border closure	Complete closure of points of entry by land, sea and/or air without specifying targeted countries/population groups/travel routes
Restricting international air travel	Stopping the arrival of international flights, restricting the origin or number of flights, rescheduling of flights, or closing airports. The measure refers to specific countries or categories of travellers
Restricting international marine travel	Stopping, restricting or rescheduling marine travel (e.g. ferries, ships). The measure refers to specific countries or categories of travellers
Restricting international land travel	Stopping, restricting or rescheduling land travel (e.g. trains). The measure refers to specific countries or categories of travellers
Individual-based travel restriction/ban	Restricting/suspending the entry/exit of specific types of travellers based on particular characteristics (e.g., immigration status, occupation, health status)
Country-based travel restriction/ban	Generally restricting/suspending international travel between a jurisdiction and one or more foreign jurisdictions
Additional travel documents	Requiring international travellers to provide certain documentation or changing arrangements of the issuance/validity of travel documents, which include, but are not limited to, a health declaration form/questionnaire, entry approval letter, visa
Other travel measures	Any other measures regulating international travel which do not fall under the measure types listed above

Source: Zhumatova S, Grépin KA, Worsnop CZ, Piper J, Song M, Lee, K. *Travel Measures and the COVID-19 Pandemic Coding Protocol*, Pandemics and Borders Project, Simon Fraser University, Burnaby, 2023

Second, this paper systematically identifies and categorizes the broad range of potential secondary outcomes using the social impact assessment framework developed by Vanclay [62]. He describes the purpose of social impact assessment as “to assess the social impacts of planned interventions or events, and to develop strategies for the ongoing monitoring and management of those impacts.” We heuristically apply five categories of social impact developed by Vanclay in this scoping review:

- way of life: how people live, work, play and interact with one another on a day-to-day basis;
- culture: shared beliefs, customs, values and language or dialect;
- community: cohesion, stability, character, services and facilities;
- personal and property rights: whether people are economically affected, or experience personal disadvantage which may include violation of their civil liberties;
- fears and aspirations: perceptions about safety, fears about the future of the community, and aspirations for the future and the future of their children.

We omit categories of political systems and environmental impacts. While politics is an important domain of the social realm, and selected studies reviewed mention political impacts, we exclude this category because based on preliminary searches, fuller treatment warrants expansion beyond Vanclay’s definition as “the extent people are able to participate in decisions that affect their lives, the level of democratisation that is taking place, and the resources provided for this purpose.” [62]. In relation to international travel measures, political systems could also include what political institutions governed their use, how public policy decisions were made, and what actors contributed to policy processes (e.g., lobbying, advisory roles). Environmental impacts are also omitted from this review, as their breadth, including across diverse contexts, and scale, also warrant separate, more detailed review. Together, our standardized taxonomy and Vanclay’s social impacts categories are used as a conceptual framework to structure this scoping review.

## Methodology

Given the new and unprecedented use of travel measures, we conduct a scoping review “as useful for examining emerging evidence when it is still unclear what other, more specific questions can be posed and valuably addressed by a more precise systematic review” [43]. Following the PRISMA-ScR approach [60], our review: a) identifies the types of available evidence on the social impacts of international travel measures; b) clarifies key concepts/definitions in the literature; c) examines how research is conducted on this topic; and d) identifies and analyses knowledge gaps.

### Search strategy

Based on the conceptualization of social impacts and taxonomy of travel measures described above, we generated keywords to search and review the existing literature, focused on travel across international borders (Table 2). We tested each potential keyword and keyword combination iteratively using Boolean terms, and refined the search when needed to minimize duplication. The search also included at least one COVID-19 pandemic keyword related to the virus in general (e.g., coronavirus, corona virus, 2019-ncov, ncov19, 2019-novel CoV, COVID, COVID-19, SARS-CoV-2), its variants and sub-variants (e.g. Alpha, Beta, Delta, Gamma, Omicron), or keyword combinations related to the virus. One reviewer (SZ) drafted the search strategy protocol and initial list of keywords and MESH terms. The list was discussed and modified by the review team (YB, KL, CW) and keywords were tested iteratively by SZ.

**Table 2:** Keywords and Search Syntax

Keyword category	Search syntax
Covid-19 (Ovid Medline)	(((exp Coronavirus/ or exp Coronavirus Infections/ or (coronavirus* or corona virus* or OC43 or NL63 or 229E or HKU1 or HCoV* or ncov* or covid* or sars-cov* or sarscov* or Sars-coronavirus* or Severe Acute Respiratory Syndrome Coronavirus*).mp.) and (201,906* or 201,907* or 201,908* or 201,909* or 20,191* or 2020* or 2021* or 2022* 2023* or 2024* or 2025* or 2026* or 2027* or 2028* or 2029* or 2030*).dt,ez,da.) not (SARS or SARS-CoV or MERS or MERS-CoV or Middle East respiratory syndrome or camel* or dromedar* or equine or coronary or coronal or covidence* or covidien or influenza virus or HIV or bovine or calves or TGEV or feline or porcine or BCoV or PED or PEDV or PDCoV or FIPV or FCoV or SADS-CoV or canine or CCov or zoonotic or avian influenza or H1N1 or H5N1 or H5N6 or IBV or murine corona*).mp.) or (Covid-19/ or covid.mp. or covid19.mp. or 2019-ncov.mp. or ncov19.mp. or ncov-19.mp.or 2019-novel CoV.mp. or sars-cov2.mp. or sars-cov-2.mp. or sarscov2.mp. or sarscov-2.mp. or Sars-coronavirus2.mp. or Sarscoronavirus-2.mp. or SARS-like coronavirus*.mp. or coronavirus-19.mp.or Deltacron.mp. or Omnicron.mp. or ((novel or new or nouveau) adj2(CoV or nCoV or covid or coronavirus* or corona virus or Pandemi*2)).mp. or ((subvariant* or variant*) adj2 (India* or "South Africa*" or UK or English or Brazil* or alpha or beta or delta or gamma or kappa or lambda or mu or "AY.X" or "BA.1" or "BA.2" or "BA.3" or "BA.4" or "BA.5" or "P.1" or "C.37")).mp. or ("B.1.1.7" or "B.1.351" or "B.1.617.1" or"B.1.617.2" or "B.1.1.529*" or "B.1.61.7*" or "21L/BA.2" or "21 K/BA.1").mp.)
Covid-19 (other databases)	covid-19 or coronavirus or 2019-ncov or sars-cov-2 or cov-19 or 2019 pandemic or pandemic
Travel measures	(border* adj3 (clos* or restrict* or control* or measure?)).ti,ab ((mobility or movement*) adj3 (reduc* or restrict*)).ti,ab (travel adj3 (measure? or intervention? or NPI?)).ti,ab ((travel* or border) adj3 (restrict* or reduc* or control* or limit* or ban*)).ti,ab (travel* adj3 (suspen* or advice or warning or advisory or screen*)).ti,ab ((isolat* or quarantin* or vaccin*) adj3 (expos*or suspect* or travel* or airport? or border?)).ti,ab travel restriction*.ti,ab
Social impacts	social interaction.ti,ab work life.ti,ab free time.ti,ab way of life.ti,ab lifestyle.ti,ab discrimination.ti,ab stigma.ti,ab loneliness.ti,ab companionship.ti,ab (shared adj (belief* or custom* or tradition* or value* or language* or or culture* or habit*)).ti,ab (belief* or custom* or tradition* or value* or language* or culture* or habit*).ti,ab (social adj (cohesion or solidarity or stability or service* or communit* or group*)).ti,ab equity*.ti,ab equality*.ti,ab divisiveness.ti,ab ((quality or availability) adj2 (air or water or water or food)).ti,ab (expos* adj2 (noise or dust)).ti,ab (environmental adj (hazard* or risk*)).ti,ab physical safety.ti,ab ((fear* or perception* or aspiration*) adj3 (safety or future or communit*)).ti,ab personal security.ti,ab

For Ovid Medline, we used a combination of COVID-19 keywords applied by Campbell’s (n.d.) search filter syntax [10]. The searches thus included a combination of three keyword categories: a) at least one COVID-19 travel measure keyword; (b) at least one COVID-19 keyword and MESH term; (c) at least one social impact keyword. In sum, the final query equals the following formula: a + b + c. For COVID-19 keywords and MESH terms, we use a multi-purpose search option that includes the title, original title, abstract, subject heading name of substance, and registry word. For keywords and keyword combinations related to international travel measures and social impacts, we searched the title, abstract, and keyword.

The search strategy was developed for Ovid MEDLINE and adapted for eight other databases. For the other eight databases we excluded some medical-specific terms for COVID-19 such as AY.X or BA.1 or BA.2 or BA.3" or BA.4 because, according to our initial keyword testing conducted in these databases, such terms were rarely used in social sciences research. For COVID-19 keywords, we thus used a simpler search combination for these databases: COVID-19 or coronavirus or 2019-ncov or sars-cov-2 or cov-19 or 2019 pandemic or pandemic.

We used Zotero to export records from databases and Covidence to manage, screen, and de-duplicate records. After de-duplication, titles and abstracts were screened by SZ and YB. The title and abstract screening were followed by a discussion of unclear cases between SZ, YB and the rest of the review team. At the next stage, the two reviewers screened the full text in duplicate and identified discrepancies and reasons for exclusion. An initial decision on inclusion was made by SZ and then finalized by the remaining authors.

### Eligibility criteria

The publication date range searched was January 2020 to December 2023 inclusive. The searches were conducted on May 22, 2023 and January 22, 2024. We searched nine databases relevant to public health and the social sciences: Ovid MEDLINE, Canadian Business and Current Affairs Database, Coronavirus Research Database, EconLit, Periodicals Archive Online, Publicly Available Content Database, Sociology Database, Sociological Abstracts Database, Applied Social Sciences Index and Abstracts (ASSIA).

For inclusion, a study needed to be: a) published in a peer-reviewed journal, book or electronic source; b) report original research based on empirical data; c) report on at least one type of international travel measure applied in response to the COVID-19 pandemic; and d) report on at least one category of social impact. The inclusion and exclusion criteria are summarized in Table 3.

**Table 3:** Inclusion and Exclusion Criteria

	Inclusion criteria	Exclusion criteria
Publication type	Published peer-reviewed journal article, book chapter or report	All other publication venues
Study design	Reports original research based on empirical data with clearly described methodology	Publications based solely on normative arguments, reviews of existing literature or evidence
Types of policy interventions	Reports on at least one type of international travel measure applied in response to the COVID-19 pandemic	Reports only on travel measures applied at a subnational level (domestically) or non travel-related measures in response to the COVID-19 pandemic Measures related to the international movement of trade (non-human animals, goods and services)
Outcomes	Reports on at least one category of social impact during the COVID-19 pandemic as defined by Vanclay [62]	Reports on only economic or health impacts
Referent groups	Individuals, populations	countries, governments, businesses (firms), organizations

### Study selection

**Fig. 1:**
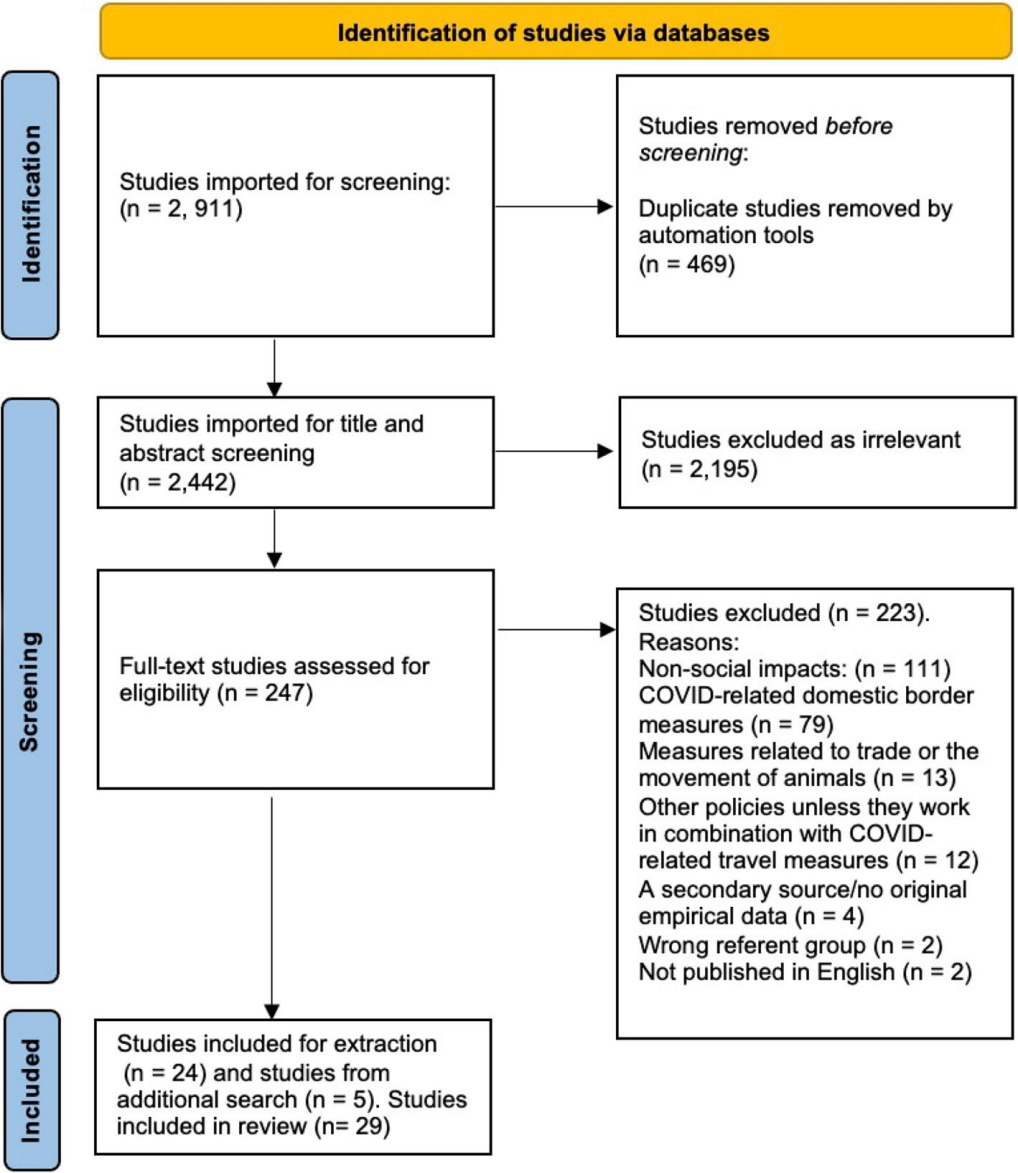
PRISMA Flow Diagram

Our search yielded 2, 911 records. After de-duplication, we identified 2,442 unique records. These were screened against title and abstract and 2,195 records were excluded. We assessed 247 studies for full-text eligibility. Five additional records were identified through citation searches. Of these, 29 studies were selected for detailed review (see Fig. 1).

### Data extraction and analysis

For the included studies, we developed and charted a summary of study characteristics using Microsoft Excel. Charting was performed across the following fields: study ID, study design and methodologies, geographical setting(s), type of travel measure based on information provided and coded by the authors, population(s) studied, category of social impact, and study period (see Table 4). This data was then collated, summarized, and reported in tabular and narrative form to identify knowledge gaps.

**Table 4:** Characteristics of studies included for the scoping review

Publication	Study setting	Study population	Study period	Study Methods	Type of international travel measure	Category of Social Impact
Avalos, M. A., & Moussawi, G. (2023). (Re)framing the emerging mobility regime at the U.S.-Mexico borderlands: COVID-19, temporality, and racial capitalism. *Mobilities, 18*(3), 408–424 [1]	United States, Mexico	crossborder commuters between the US and Mexico	March 2020—December 2020	qualitative document analysis, social media analysis	travel restrictions (entry/exit from Mexico limited to ‘essential workers’)	community (solidarity, support networks online), personal and property rights (employment), fears and aspirations (uncertainty about COVID-19 restrictions)
Brooks, S. K., Patel, D., & Greenberg, N. (2023). Exceptionally challenging time for all of us: Qualitative study of the COVID-19 experiences of partners of diplomatic personnel. *PloS One, 18*(11), e0293557-e0293557 [7]	United Kingdom, Asia, Africa, Europe, Oceania, North America	Partners of British diplomats (n = 11)	September 2021—January 2022	qualitative interviews (10 by video conference, 1 in person)	travel restrictions (entry limited to citizens and residents), quarantine, testing	way of life (childcare), community (communication, solidarity, support networks), personal and property rights (employment), fears and aspirations (infection)
Campbell, N., Thompson, S. C., Tynan, A., Townsin, L., Booker, L. A., & Argus, G. (2021). Silver linings reported by Australians experiencing public health restrictions during the first phase of the COVID-19 pandemic: A qualitative report. *International Journal of Environmental Research and Public Health*, 18(21), 11,406 [9]	Australia	Australian residents (n = 90)	August—December 2020	qualitative interviews (telephone and video conference)	travel restrictions (entry limited to citizens and residents)	community (resilience, solidarity), fears and aspirations (infection)
Eslami, F, Namdar, R. (2022). Social, Environmental and Economic Impact Assessment of COVID-19 on Rural Tourism. *Frontiers in Public Health*, 10: 883,227 [15]	Iran	Local residents (n = 235) and tourism sector stakeholders in Natanz City (n = 110)	n.d	quantitative survey	travel restrictions (exit limited to essential travel in source countries)	culture (impacts of tourism), community (solidarity), personal and property rights (employment)
Fatmi, M. R. (2020). COVID-19 impact on urban mobility. *Journal of Urban Management*, 9(3), 270–275 [16]	Canada	Residents of Kelowna, British Columbia (n = 202)	March -May 2020	Quantitative survey	travel restrictions (entry limited to citizens and residents)	way of life (travel behaviours)
Golunov, S., Smirnova, V. (2022). Russian border controls in times of the COVID-19 pandemic: Social, political, and economic implications. *Problems of Post-Communism*, 69(1), 71–82 [18]	Russia	Immigrants from former Soviet Union countries	January – August 2020	Qualitative document analysis	travel restrictions (entry and exit initially targeted countries; entry later limited to diplomats, permanent residents, transit passengers, relatives of deceased, transport personnel; additional categories of exemption added), flight bans from selected source countries, border closure (exit by Russian citizens banned), screening, quarantine for inbound travellers	way of life (family relationships), community (privileged access to travel, stigma), personal and property rights (employment)
Guillen-Royo, M. (2022). Flying less, mobility practices, and well-being: Lessons from the COVID-19 pandemic in Norway. *Sustainability: Science, Practice, & Policy*, 18(1), 278–291 [22]	Norway	Survey of representative adults in Norway (n = 2019) and interviews with adults in Norway with experience of travel between Oslo and Bodø (n = 12)	survey in autumn 2017, interviews between February—September 2020	Mixed methods: survey (on-line), interviews (9 by video conference, 3 in person)	travel restrictions (entry limited to returning citizens and residents), quarantine for non-essential travellers	way of life (travel behaviours), culture (cultural capital, social norms)
Hari, A, Nardon L, Zhang H. A transnational lens into international student experiences of the COVID19 pandemic. (2023). *Global Networks*, 23: 14–30 [23]	Canada	international students in Ontario (n = 13)	April – June 2020	Qualitative interviews (video conference)	travel restrictions (entry limited to essential travel)	way of life (day-to-day life, family relationships), personal and property rights (employment, opportunity), fears and aspirations (infection)
Holleran, M. (2022). Pandemics and geoarbitrage: Digital nomadism before and after COVID-19. *City*, 26(5–6), 831–847 [24]	Low- and middle-income countries	“digital nomads in low and middle-income countries (n = 25)	2020	Qualitative interviews (telephone), social media analysis	travel restrictions (unspecified)	community (connection, services), personal and property rights (employment, opportunity)
Hu, Y., Xu, C. L., & Tu, M. (2022). Family-mediated migration infrastructure: Chinese international students and parents navigating (im)mobilities during the COVID-19 pandemic. *Chinese Sociological Review*, 54(1), 62–87 [25]	United Kingdom	Chinese international students in UK (n = 16) and student parents in China (n = 8)	April—May 2020	Qualitative interviews (on-line)	Evolving restrictions to international and domestic mobility in UK; border controls, quarantine and reduced flights in China	way of life (travel behaviour, family relationships), community (privilege), personal and property rights (employment), fears and aspirations (personal safety)
Ittmann, H.W. (2022). The impact of COVID-19 on informal humanitarian supply chains – the case study of Zimbabwe. *Journal of Transport and Supply Chain Management*, 16(1), e1-e15 [28]	Zimbabwe, South Africa	Zimbabwe diaspora in South Africa	2020	Mixed methods: secondary data, interviews, interactions and discussions (in person)	travel restrictions (entry by land limited to commercial cargo, diplomats, bodies for burial, light commercial vehicles, and citizens and residents)	way of life (family relationships), personal and property rights (humanitarian assistance)
Kelly, C. (2022). ‘I just want to go home’: Emotional wellbeing impacts of COVID-19 restrictions on VFR travel. *Tourism and Hospitality*, 3(3), 634–650 [30]	United Kingdom	UK-based diaspora unable to travel home during COVID-19 (n = 70)	December 2020—March 2022	Qualitative interviews (in person and on-line) and survey (on-line)	travel restrictions (unspecified)	way of life (travel behaviour), culture (identity), community (diaspora, belonging), fears and aspirations (infection, family well-being)
Kupi, M., & Szemerédi, E. (2021). Impact of the COVID-19 on the destination choices of Hungarian tourists: A comparative analysis. *Sustainability*, 13(24), 13,785 [32]	Hungary	Hungarian residents who had travelled at least once a year for holidays (n = 500)	May—July 2021	Quantitative survey	travel restrictions – lifting (unspecified)	way of life (travel behaviour)
Lan P.C. (2022). Shifting borders and migrant workers’ im/mobility: The case of Taiwan during the COVID-19 pandemic. *Asian and Pacific Migration Journal*, 31(3): 225–246 [33]	Taiwan	Migrant workers from the Philippines and Indonesia with experience of constraints to labour mobility (n = 27)	May – October 2021	Qualitative interviews (22 video conference, 5 in person)	travel restrictions (entry limited to diplomats, foreign students, business travelers and holders of valid Alien Resident Certificates), quarantine (mandatory 14 days at designated site), digital tracking system Other countries: travel restrictions (delayed or non-issuance of visas, flight cancellations)	way of life (independence), personal and property rights (employment), fears and aspirations (infection)
Leiblfinger, M., Prieler, V., Rogoz, M., & Sekulová, M. (2021). Confronted with COVID-19: Migrant live-in care during the pandemic. *Global Social Policy*, 21(3), 490–507 [36]	Austria, Romania, Slovakia	Romanian and Slovakian live-in care workers in Austria; Austrian-based care sector stakeholders (n = 4)	March—September 2020	Qualitative interviews (mode unspecified), media analysis, policy analysis	travel restrictions (entry limited to citizens and residents) with exemptions through transit and care corridors, quarantine and/or testing by Austria; quarantine in government facility and home, and testing of returning care workers by Slovakia	community (solidarity), personal and property rights (employment), fears and aspirations (patient well-being)
Liu, L. S., Ran, G. J., & Jia, X. (2022). New Zealand border restrictions amidst COVID-19 and their impacts on temporary migrant workers. *Asian and Pacific Migration Journal*, 31(3), 312–323 [38]	New Zealand	migrant workers from Pacific Island countries	March 2020—February 2022	Mixed methods: secondary data analysis, media analysis and scholarly literature	travel restrictions (entry limited to citizens and residents)	way of life (access to workplace abroad), culture, personal and property rights
McDermid, P., Craig, A., Sheel, M., Blazek, K., Talty, S., & Seale, H. (2022). Examining the psychological and financial impact of travel restrictions on citizens and permanent residents stranded abroad during the COVID-19 pandemic: International cross-sectional study. *BMJ Open*, 12(5), e059922-e059922 [40]	Europe, Western Pacific Region	Self-identified individuals stranded abroad during COVID-19 pandemic due to international travel restrictions (n = 1054)	January 2020 – September 2021	Quantitative survey	travel restrictions, border closures	personal and property rights (employment, housing, human rights)
Melov, S. J., Galas, N., Swain, J., Alahakoon, T. I., Lee, V., Cheung, N. W., McGee, T., Pasupathy, D., & McNab, J. (2022). Exploring the COVID-19 pandemic experience of maternity clinicians in a high migrant population and low COVID-19 prevalence country: A qualitative study. *Women and Birth*, 35(5), 493–502 [42]	Australia	Midwives and medical staff of tertiary hospital (n = 14)	November – December 2020	Qualitative interviews (13 in person, 1 via videoconference)	travel restrictions (entry limited to citizens and residents) and quarantine for all inbound arrivals	way of life (family relationships), culture (family support), fears and aspirations (patient well-being, health worker well-being)
O'Connor, C., O'Connell, N., Burke, E., Dempster, M., Graham, C. D., Scally, G., Zgaga, L., Nolan, A., Nicolson, G., Mather, L., Barry, J., Crowley, P., & Darker, C. D. (2021). Bordering on crisis: A qualitative analysis of focus group, social media, and news media perspectives on the Republic of Ireland-Northern Ireland border during the ‘first wave’ of the COVID-19 pandemic. *Social Science and Medicine* (1982), 282, 114,111–114,111 [45]	Ireland, Northern Ireland	Communities living near Ireland-Northern Ireland border	December 2019 – August 2020	Qualitative focus groups (video conference), news analysis, social media analysis	travel restrictions (exit and entry limited to essential travel)	way of life (crossborder lifestyle), community (cohesion, conflict, ethnocentrism), personal and property rights (employment), fears and aspirations (infection, community well-being)
Olani, A. B., Degefa, N., Aschalew, Z., Kassa, M., Feleke, T., Gura, G., & Wambete, S. N. (2022). Exploring experiences of quarantined people during the early phase of COVID-19 outbreak in southern nations nationalities and peoples’ region of Ethiopia: A qualitative study. *PLoS One*, 17(9), e0275248-e0275248 [46]	Ethiopia	adults (≥ 18 years) who stayed in a quarantine center in Southern Nations, Nationalities and People’s Region (n = 29)	June—July 2020	Qualitative interviews (in person)	Quarantine (mandatory 14-days in designated site for inbound travellers at their expense)	way of life (day-to-day life), culture (response to virus and quarantine), personal and property rights (employment, human rights), fears and aspirations (infection, family well-being)
Peyrony, J., Rubio J, Viaggi R. *The effects of COVID-19 induced border closures on cross-border regions*. Luxembourg: Mission Opérationnelle Transfrontalière (MOT) and European Union, 2021 [49]	European Union members	Stakeholders related to transport and border regions (n = 21)	July – August 2020	Qualitative interviews (mode unspecified)	travel restrictions (entry and exit limited by varied categories including citizenship, purpose of travel etc.)	way of life (travel behaviour, family relationships), culture (events), community (trust, stigma, discrimination, social inclusion, belonging), personal and property rights (employment), fears and aspirations (border security)
Qiu, R., Park J, Li SN, Song HY. (2020). Social costs of tourism during the COVID-19 pandemic. *Annals of Tourism Research*, 84: 102,994 [51]	China	Residents of Hong Kong, Guangzhou and Wuhan, China (n = 520)	February – March 2020	Quantitative survey	travel restrictions (unspecified)	fears and aspirations (community well-being, safety)
Raja, R., Ma, J., Zhang, M., Li, X. Y., Almutairi, N. S., & Almutairi, A. H. (2023). Social identity loss and reverse culture shock: Experiences of international students in China during the COVID-19 pandemic. *Frontiers in Psychology, 14*, 994,411–994,411 [52]	China and unspecified countries of Global South and Eastern Europe	International students who remained in China during the pandemic or were stranded in their home countries (n = 20)	April 2020—March 2021	Qualitative interviews (video conference), social media analysis	border closure (China)	culture (reverse culture shock), community (social networks, group membership, discrimination), personal and property rights (study and work disruptions)
Refaat, S. A., Arafa, H. F. (2022). Investigating the effect of COVID-19 global travel restrictions on tourists’ travel behavior, habits and intentions “applied study on Saudi tourists”. *GeoJournal of Tourism and Geosites*, 40(1), 49–55 [53]	Saudi Arabia	Saudi citizens travelling abroad for holiday on first two days of official permission granted to travel abroad (n = 450)	May 2021	Quantitative survey	travel restrictions (exit limited to essential travel)	way of life (travel behaviours), culture (religion), fears and aspirations (infection)
Schneiders, M. L., Naemiratch, B., Cheah, P. K., Cuman, G., Poomchaichote, T., Ruangkajorn, S., Stoppa, S., Osterrieder, A., Cheah, P., Ongkili, D., Pan-Ngum, W., Mackworth-Young, C. R. S., & Cheah, P. Y. (2022). The impact of COVID-19 non-pharmaceutical interventions on the lived experiences of people living in Thailand, Malaysia, Italy and the United Kingdom: A cross-country qualitative study. *PLoS One*, 17(1), e0262421-e0262421 [55]	Thailand, Malaysia, Italy, United Kingdom	Purposive sample of survey respondents who are residents of Thailand, Malaysia, Italy, United Kingdom (n = 86)	May – August 2020	Qualitative interviews (email and telephone)	travel restrictions (amid wide range of NPIs)	way of life (family relationships), community (solidarity, connection), personal and property rights (employment), fears and aspirations (infection, healthcare workers, family well-being)
Simola, A., May, V., Olakivi, A., & Wrede, S. (2023). On not ‘being there’: Making sense of the potent urge for physical proximity in transnational families at the outbreak of the COVID‐19 pandemic. *Global Networks*, 23(1), 45–58 [56]	Finland, Belgium	migrants living in Finland and Belgium with family members (≥ 65 years) living in another country (n = 41)	April—June 2020	Qualitative written narratives	Finland: travel restrictions (entry limited to citizens and residents) Belgium: travel restrictions (exit and entry limited to essential travel) Quarantine (mandatory upon entry for inbound travellers)	way of life (family relationships), personal and property rights, fears and aspirations (family well-being)
Skovgaard-Smith I. (2021). Transnational life and cross‐border immobility in pandemic times. *Global Networks*, 23(1), 59–74 [57]	26 countries	Transnational professionals via researcher’s social networks (interviews n = 36; survey n = 102)	May 2020–May 2021	Qualitative interviews (video conference), survey (on-line)	travel restrictions in 26 countries (entry and exit limited under varied categories)	way of life (travel behaviour), community (isolation), personal and property rights (employment)
Tarvet, R., & Klatt, M. (2023). The impact of the corona crisis on borderland living in the Danish-German border region with a special focus on the two national minorities. *National Identities*, 25(1), 35–52 [59]	Denmark, Germany	Members of national minority communities living in Danish-German border region of Schleswig (n = 392)	July—August 2020	Quantitative survey	travel restrictions (entry and exit via land borders limited to exempted categories), quarantine, testing	way of life (travel behaviour, family relationships), community (connection)
Zentveld, E., Erol, G., & Düşmezkalender, E. (2022). VFR travel in Turkey during and post-COVID-19. *Tourism and Hospitality*, 3(3), 651–665 [65]	Turkey	Turkish residents (n = 22)	December 2021—January 2022	Qualitative interviews (in person and telephone)	travel restrictions	way of life (travel behaviour), culture (importance of travel), fears and aspirations (community well-being)

## Results

### Study setting

Twenty-seven (93%) of the reviewed studies focus on national settings, either by individual country, regional groups of countries, or comparative analysis of countries. The studies also spanned a diverse range of national settings as a reflection of the near-universal use of international travel measures during the COVID-19 pandemic. Sixteen studies focused on a population within a single country: Australia [9, 42], Canada [16, 23], China [51], Ethiopia [46], Hungary [32], Iran [15], New Zealand [38], Norway [22], Russia [18], Saudi Arabia [53], Taiwan [33], Turkey [65], and the United Kingdom [25, 30]. Given international travel concerns human mobility between countries, six studies focus on how travel measures impacted populations across two or more national settings: the United States and Mexico [1], Zimbabwe and South Africa [28], Austria, Romania and Slovakia [36], Finland and Belgium [56], Ireland and Northern Ireland [45], and Denmark and Germany [59]. Five studies analyzed the social impacts of travel measures within a region or across regions: European Union [49], WHO European and Western Pacific Regions [40], Asia, Africa, Europe, Oceania, North America [7], China, Eastern Europe and the Global South [52], and Thailand, Malaysia, Italy and the United Kingdom [55].

We reviewed two studies which are exceptions to state-centric analyses of (im)mobility [24, 57]. These studies begin with recognition of new spatial logics arising from processes of economic globalization. Since the 1990s, globalization has not only exponentially increased the intensity and extensity of population mobility, but has created new “transnationalized” patterns of mobility [13, 17]. The use of travel measures during the COVID-19 pandemic has disrupted many forms of transnational migration. Holleran [24] documents how travel measures have challenged the core practices of “digital nomads,” individuals “working [virtually] for higher wages in developed countries but living in less expensive locations, most often in the Global South.” Motivated by an “elite cosmopolitan” outlook, nomads from high-income countries previously favoured visa-free open borders, eschewing “a relationship with the state in destination countries for taxation reasons.” When the pandemic led to increased health risks alongside prolonged mobility restrictions, the digital nomads interviewed describe themselves as reevaluating their relationships with their home and resident states. Holleran reports an increased appreciation for the state, not simply as an imposer of mobility restrictions, but as provider of health care and other essential services. Similarly, Skovgaard-Smith [57] explores the “impact of prolonged, involuntary cross-border immobility on transnational lives in the context of a global crisis.” Based on experiences ranging from privileged “transnational professionals” to more precarious “transnational migrants”, the study gathers varied accounts of how “ways of being” and “ways of belonging” are disrupted by abrupt disruption to “intensely transnationalized lives” dependent on international travel to maintain family relationships and fulfil social obligations prior to the pandemic.

### Study population

The studies reviewed focus on diverse populations impacted by international travel measures. Fourteen studies (48%) examined the experiences of residents in their home countries who were unable to travel abroad due to exit and/or entry restrictions. Six of these studies focused on changes in travel behaviours largely related to tourism such as mode of transport and choice of holiday destination [15, 16, 32, 51, 53, 65]. Five of the studies were concerned with the broader social impacts of travel restrictions on residents [9, 22, 55], including two studies on the particular experiences of border communities [45, 59]. Three of the studies focused on resident populations defined by lived experiences: midwives and health care workers in Australia [42], transport sector stakeholders in the European Union [49], and adults who quarantined in Ethiopia [46].

Fifteen studies (52%) analysed the experiences of migrant populations based outside of their home country/country of origin who were limited in their ability to travel internationally. These included migrant workers in New Zealand [38], Taiwan [33], and Austria [36]; transborder commuters at the U.S.-Mexico border [1]; immigrants in Russia [18], Finland and Belgium [56]; partners of British diplomatic personnel [7]; international students in Canada [23], China [52], and the UK [25]; diaspora in South Africa [28] and the UK [30]; and other foreign nationals [24, 40, 57].

Finally, three studies also analyzed the social impacts resulting from interactions between residents and migrant populations: Chinese international students in the UK and their parents in China [25], Zimbabwean residents and diaspora in South Africa [28], and migrant care workers from Romania and Slovakia working in Austria, and care sector stakeholders in all three countries [36].

### Study period

Seventeen studies conducted data collection only during the first year of the pandemic (December 2019-December 2020) or collected data relevant to this period. This was when virtually all countries rapidly introduced screening and, most impactfully, travel restrictions that severely limited the entry and exit of travellers into their jurisdictions. This is thus when the social impacts of travel restrictions were likely to have been experienced most acutely, and thus a prompt for their study. As the pandemic continued, many governments adjusted restrictions to allow some exemptions, such as new categories of “essential travel”, or lifted restrictions temporarily on non-essential travel. However, other travel measures (i.e., testing, quarantine and vaccination requirements) were introduced for travellers by many countries which remained in place, and were frequently adjusted, over the next two years. The study periods of ten studies reviewed extend into 2021 and 2022 to analyze this prolonged use of travel measures. One study compared a period before and then during the pandemic to measure changes in travel behaviours [22]. One study did not report a study period [15].

### Study methods

Nineteen studies used qualitative methods and seven studies used quantitative methods, with three studies applying mixed (qualitative and quantitative) methods. However, the conduct of in-person research on human subjects during the COVID-19 pandemic was severely hindered by public health orders in most countries to reduce the risk of virus transmission including social distancing and lockdowns. Thus, what and how methods were applied reflected the need to comply with these requirements.

All seven quantitative studies used the survey method to gather data on travel behaviours, intentions, and attitudes. For these studies, the conduct of surveys was almost exclusively through on-line platforms (Fatmi, 2020). While this does not seem to have had a detrimental effect on the quality of the responses obtained, given the common use of on-line platforms in many countries to conduct surveys, several studies noted sampling challenges. For example, Kupi and Szemerédi drew upon the existing Hungarian National Panel used for marketing and sociological research which was acknowledged as not meeting “the criterion for representativeness.” [32]. Similarly, Tarvet and Klatt report limited “representativity of the survey regarding the whole population of the border region,” and a “natural bias resulting from a higher probability of respondents personally affected by the border closure.” [59] Sampling was a particular challenge for Eslami and Namdar which was the sole quantitative study to collect responses in-person to a questionnaire in a tourist region of Iran. The authors describe “restrictions on the spread of the corona virus, access to tourists, locals and data collection, as well as travel to tourist areas faced difficulties.” [15]

Of the nineteen qualitative studies and two mixed methods studies, seventeen use interviews to gather experiences of social impacts arising from international travel measures. Other methods applied were media analysis (6), document and policy analysis (3), focus groups (1), personal communications (1) and written narratives (1). Given constraints on conducting in-person research on human subjects, along with the disparate locations of participants for several studies, interviews were conducted online by seven studies, in-person by two studies, and a combination of both by six studies. The focus groups used by one study were conducted online [45].

The increased need for virtual (online) data collection potentially poses a particular challenge for qualitative researchers. As Skovgaard-Smith writes, “in the middle of a pandemic, there were no physical field sites to enter or places to go to, only fragmented virtual spaces.” [57] For instance, ethnographic research aims “to observe and analyze how people interact with each other and with their environment in order to understand their culture” [14]. Similarly, a phenomenological approach “seeks to describe the essence of a phenomenon by exploring it from the perspective of those who have experienced it. The goal of phenomenology is to describe the meaning of this experience—both in terms of *what* was experienced and *how* it was experienced” [44]. Such approaches are necessary for the study of the social impacts of international travel measures but they demand deep engagement, detailed observation, and contextualized understanding of the experiences and perceptions of study participants. Kelly [30], for example, sought to analyze the “emotional impacts of restricted access, to core pillars of identity, love and belonging, along with emotional/wellbeing implications and respondents’ adopted coping strategies of restricted travel.” Schneiders et al. [55] adopted a phenomenological approach “to explore and compare the lived experiences, coping strategies and views of government imposed COVID-19 NPIs among the public and HCW [healthcare workers].”

Aware of the limitations of online data collection, a few studies developed innovative methods as supplemental or even alternatives to in-person fieldwork. For example, Skovgaard-Smith [57] conducted a “virtual ethnographic study” through 36 interviews and administered 102 surveys using “Zoom, MS Teams, WebEx, Whatsapp or Skype.” Beginning with the principle of “transformative interviewing,” which “rejects the notion that interviews are neutral activities in which knowledge is transferred from participants to researchers,” Hari et al. [23] experimented with two interventional techniques to facilitate reflection: photo elicitation technique and integral coaching conversations. Six studies drew upon media analysis given the key role of mainstream and social media in reporting the lived experiences of people worldwide during the pandemic. The further development of these methods, and their validation against qualitative research unhindered by pandemic restrictions, will be important for their future use.

### Type of international travel measure

The terms most frequently used by the studies reviewed, when referring to international travel measures, were border closures [22, 23, 52, 55] and border controls [9, 18]. Strictly speaking, however, most governments did not close their borders, but instead restricted who could cross them (e.g., citizens and permanent residents) and/or for what purpose (e.g., essential travel). Moreover, while borders are focal points for entry to and exit from the territories of sovereign states, during the COVID-19 pandemic, population mobility was disrupted before, at and after actual border crossings. For this reason, we use the term “travel measure” to describe interventions used to manage who can travel and for what purposes during the pandemic, and distinguish among many types of travel measures including screening, restrictions, quarantine, testing and health/immunity certification. For this scoping review, we applied this standardized typology and terminology to accurately describe the travel measure analyzed in each article.

Twenty-six of the studies (90%) analysed travel restrictions which involved limitations to entry into and/or exit from a national jurisdiction by certain categories of travellers or for selected purposes of travel. This may be due to 21 studies (72%) focussing on the first year of the pandemic when such restrictions were rapidly and widely introduced, and perhaps most disruptive to people’s lives. Despite frequent use of the term border closure, only three studies analyse this type of travel measure [18, 40, 52].

Where travel restrictions permitted entry into a country, ten studies analyzed the social impacts of quarantine requirements. These requirements varied significantly by who was subject to quarantine, mandatory length, stipulated location, conditions and cost. Four studies discuss the screening (including testing) of travellers [7, 18, 36, 59], three studies analyze the reduced availability of flights [18, 25, 33], and one study considers the requirement for additional travel documents [33].

Importantly, many studies analyse the social impact of international travel measures as part of a broad range of public health measures applied by countries including domestic travel restrictions, social distancing, and lockdowns. For example, Campbell et al. ( [9], pp. 1 – 2) study Australian experiences of “closure of international borders, state and territory border closures, limitations on the movement and numbers allowed at social gatherings, closure of businesses with only those deemed essential allowed to stay open, and the requirement for people to work from home where possible.” Fatmi ([20, p. 274) defines “long-distance travel during the COVID-19 travel restrictions” as “*regional travel* which is travel within the same province or state, *domestic travel* which is travel within the same country, and *international travel* which is travel across borders.” In addition, while most studies sought to analyse the social impacts of international travel measures at a given point in time, in practice, the use of these measures was highly changeable. This creates challenges for assessing what specific travel measures, and how they were implemented, had the assessed outcomes.

Overall, international travel measures are described with limited specificity in the studies reviewed, often with imprecise and inconsistent terminology, or grouped together with other public health measures. While the impacts of domestic and international travel measures may be interrelated, where better understanding of the social impacts arising from international travel measures is sought, to inform future decision making, this raises the problem of findings resulting from conflation or confounding by other policy measures taken during the COVID-19 pandemic. Future studies should clearly distinguish what policy measures are being studied.

### Category of social impact

While there is widespread recognition and growing study of the secondary outcomes arising from responses to the COVID-19 pandemic, there is no agreed framework for categorising these outcomes. In this review, we apply the social impact assessment framework categories by Vanclay to synthesize the reviewed studies [62].

#### Way of life

Twenty-one of the 29 studies reviewed (72%) reported impacts from travel measures on way of life in their findings. Hari et al. [23] document the “daily enactment of transnationalism” by international students, and the “interrupted access to transportation, housing insecurity, precarious and/or temporary immigration status and unemployment” caused by travel restrictions. Similarly, Ittmann [28] analyses the adverse impact on food security of Zimbabweans dependent on the assistance of diasporic relatives in South Africa. Olani et al. [46] study the impact on day-to-day life of inbound travellers to Ethiopia subject to a 14-day quarantine. Brooks et al. [7] reported disruptions associated with the caregiving of children among families of British diplomatic personnel.

One particularly important impact on way of life was family separation which was reported by a diverse range of population groups including immigrants, transnational families, international students, and cross-border communities Golunov and Smirnova [18] and Simola et al. [56], for example, reported “not being there” for aging and ailing relatives living in another country. Tarvet and Klatt found reduced opportunities for “kin-state contact and interaction” by minority populations along the Danish-German border [59]. Schneiders et al. [55] described “separation, isolation and grief over missed milestones” from travel restrictions and quarantine in four countries. Hu et al. [25] found similar challenges facing international students in the UK, but also how “family-mediated infrastructure” was successfully mobilized to enable some students to return to China.

Finally, nine studies of impacts on way of life focus on changes in travel behaviours for by tourists, international students and foreign workers [16, 22, 25, 49]. For communities near international borders, cross-border travel was a common part of day-to-day life that was disrupted [45, 59]. The “open borders” travel patterns of “digital nomads” were also abruptly curtailed by restrictions on the entry and exit of travellers by many countries [57]. Recreational travel was deemed “non-essential” by many countries and thus significantly reduced [30, 32, 53, 65].

#### Culture

Six studies report impacts on cultural practices by travel measures during the COVID-19 pandemic. Tarvet and Klatt describe disruptions to the “special culture and the shared life” that has evolved over time within border communities between Denmark and Germany [59]. The disruption to building and sustaining a shared European identity, from the cancellation of cultural events, in part due to travel measures, is described by Peyrony et al. [49]. Raja et al. describe severe reverse culture shock experienced by international students who had left China during the pandemic and were stranded in their home countries due to border closures [52].

Melov et al. document the disruption to cultural norms regarding childbirth and postpartum support for migrant women in Australia from extended family who must travel from overseas [42]. Similarly, the social impact from the inability to engage in “visiting family and friends,” as “a key part of Turkish culture”, is described by Zentveld et al. [65]. By contrast, focusing on a tourist region in Iran, Eslami and Namdar ( [15], p. 9] find the decline in tourists reduced “negative social effects such as the destruction of cultural customs and traditions.”

Two studies examined the role of culture in how people respond to travel measures. Olani et al. find that “cultural background” influenced how study participants responded to the uncertainties regarding SARS-CoV-2 and the experiences of quarantine in Ethiopia [46]. Guillon-Royo describes the influence of “cultural capital” in shaping adaptations to travel behaviour and changing social norms regarding virtual social engagement [22].

#### Community

Fourteen studies report social impacts related to community. Most describe negative impacts arising, first, from the reduced ability to travel. International travel is described as central to sustaining connections and a sense of belonging within communities [30, 49, 55, 57] and between communities straddling borders [45, 59]. Second, negative impacts on society can arise from how travel measures are implemented, with the targeting of foreign countries or migrant populations leading to stigma, discrimination and marginalization. For example, O’Connor et al. describes disruption to travel between Ireland and Northern Ireland as threatening hard fought community cohesion and encouraging a return to ethnocentrism [45]. In Russia, Golunov and Smirnova describe the further marginalization of foreign migrant populations including stranded foreign workers living in temporary camps and becoming the target of extortion by police officers [18].

The theme of solidarity was raised in different ways in the reviewed studies. Avalos and Moussawi describe how travel restrictions at the U.S.-Mexico ports of entry facilitated the use of support networks on Facebook to navigate new commuting conditions [1]. Campbell et al. report on positive community impacts from travel restrictions in Australia as “silver linings”, providing evidence of “community resilience”, and a “renewed sense of community and togetherness” arising from feelings of trust, support and solidarity [9]. The range of “inadvertent positive experiences” including “a greater sense of community” was also reported by Schneiders et al. [55]. However, Leiblfinger et al. describe how appeals to solidarity were used by employers to pressure migrant live-in care workers to resume their positions in Austria [36]. Initial feelings of solidarity were also undermined over time by the privilege of economically and politically advantaged groups in Russia [18] and wealthy international students in the UK [25] who were able to effectively circumvent travel restrictions. There was a strong sense of “othering” of foreign nationals and migrant populations where governments emphasized external risks of contagion [25, 49].

#### Personal and property rights

The main impact to personal and property rights identified concerns regarding disruptions to employment from travel restrictions and quarantine requirements. Cross-border workers were directly impacted between South Africa-Zimbabwe [28], Ireland-Northern Ireland [45], the United States and Mexico [1], and within the European Union [49]. Many migrant workers lost their jobs [33] and some were stranded abroad, such as in Russia [18], or forced to accept more challenging workplace conditions such as in Austria [36]. Employment in the tourist sector was particularly impacted [15, 49]. For “digital nomads” and other workers not tied to a specific workplace, travel measures impacted the ability to pursue employment worldwide [24, 57]. The curtailment of training and employment opportunities of international studies was addressed by two studies [23, 25].

In some cases, studies found positive effects on environment. Guillen-Royo describes an improved work-life balance for some Norwegians who eliminated work trips overseas due to travel restrictions [22]. Schneiders et al. also report “more time at home to focus on family, oneself and the essential.” [55]. Some “digital nomads” living in the Global South welcomed travel restrictions as a reason to remain in situ, away from more stringent lockdowns elsewhere, and in countries with potentially lower prevalence of COVID-19 infections [24].

Finally, there were concerns raised about potential violation of human rights from the use of travel measures. Inbound travellers required to quarantine in Ethiopia reported intimidation, harsh treatment, injustice, and detention [46]. McDermid et al. ( [40], p. 6) identify “high level of financial distress (64.2%), employment changes (38.4%) and experiences of homelessness (12%)” among surveyed populations stranded abroad due to “flight changes and delays (incurring additional costs)”. The resultant feelings of helplessness, abandonment by home and host countries, and even dehumanization is similarly reported by Skovgaard-Smith [57]

#### Fears and aspirations

Fears of SARS-CoV-2 infection was a major theme in the studies reviewed. Changes in circumstances caused by travel measures, such as loss of employment or educational opportunities, worsened working conditions or housing put some populations at greater risk of exposure to the virus (i.e., migrant workers, international students) [23, 25, 33]. O’Connor et al. and Olani et al. raise fears of infection from how travel measures were implemented by creating new vulnerabilities to virus exposure [45, 46]. Other studies describe how travel measures are seen to reduce infection risks in Australia [9] and tourist destinations [51, 53].

Fears about the health and well-being of families and communities were identified in several studies. For example, Kelly and Simona et al. described the anguish of “not being there” for family members in need by migrants stranded abroad [30, 56]. Zentveld et al. report concerns about community well-being in Iran from the sharp decline in tourism [65]. The inability of extended family from overseas to provide support to expectant mothers raise fears for the health and well-being of patients and overworked clinicians in Australia [42]. Fears for patient well-being is also raised by Leiblfinger et al.’s study of foreign live-in care workers in Austria [36].

Finally, Peyrony et al. described fears of increased cross-border criminal and terrorist activity at EU internal and external borders due to travel restrictions [49]. Fears for personal safety among Chinese international students [25] are described amid increased stigmatization of selected racialized populations targeted by international travel measures.

## Discussion

Growing support for risk-based approaches in the future use of international travel measures during PHEICs, by WHO and other international organizations, call for fuller understanding of both their public health effectiveness and broader secondary outcomes. This scoping review of evolving evidence on the social impacts of international travel measures thus complements the evolving evidence on their public health effectiveness [19]. The findings of this review raise implications for strengthening this evidence base through future research and integrating this evidence into risk-based approaches.

On study setting, social impacts are identified in a broad range of countries and regions spanning high-, middle- and low-income settings. However, only two studies focus on low-income countries – Zimbabwe/South Africa [28] and Ethiopia [46] – despite the near universal use of international travel measures and the global interconnectedness of mobile populations. Moreover, with the pandemic setting back almost all the Sustainable Development Goals, international travel measures intended to protect public health have also had, for example, “major negative impacts in countries with a high share of GDP coming from tourism and service industries” [37] There is a need for detailed case studies, of varied regional, national and local settings.

Two studies go beyond the territorial state to understand social impacts related to spatial logics associated with economic globalization and “transnational life” [24, 57]. Further study is needed to understand how these new forms of territoriality are impacted by international travel measures, and how more equitable access to them can potentially mitigate adverse social impacts (e.g., virtual workspaces). Moreover, there is a need to understand how travel measures adopted individually by national governments, in an increasingly “transnationalized” world, are likely to overlook the social circumstances of highly mobile transnational populations or those who fall between the cracks of international travel (e.g., unofficial migrants). Both point to the need for better coordinated use of travel measures by states.

There was a broad range of populations covered by the reviewed studies. Mobile populations identified include migrant workers, digital nomads, international students, diaspora, cross-border commuters and tourists. More research is needed on additional populations that travelled despite the pandemic such as transport workers (e.g., flight crew, truck drivers, seafarers), refugees and asylum seekers, and health care workers. Other studies focus on populations impacted by disrupted international travel such as patients, extended family, tourism operators, and border communities. While studies that surveyed majority populations accounted for variation in age, status, gender and other sociodemographic characteristics, equity-deserving groups within the cohort of country nationals, such as low-income earners and people with disabilities, have received little scholarly attention which suggests a significant research gap. There is a need for studies that focus on a wider range of social groups, through an intersectional lens taking account of age, status, gender and other sociodemographic characteristics.

Importantly, the studies reveal how some populations could navigate international travel measures, and thus mitigate adverse social impacts, more effectively due to their professional, political or financial status. Other populations, such as migrant workers and some international students, experienced a loss of control over their living and working conditions, and fulfilling their basic needs. For example, migrant workers who lost employment or legal status in their residence countries were forced to live in precarious conditions, limiting access to housing, healthcare, and social protections. There were few “silver linings” experienced by these populations, many of which became subject to stigma, racism and abuse. There is need to better understand the equity impacts of international travel measures on diverse populations. As O’Connor et al. conclude, “decisions to restrict cross-border movement should consider whether this may disproportionately disadvantage certain groups.”( [45], p. 2).

On study period, most focused on the social impacts experienced during the first year of the pandemic when travel restrictions were rapidly introduced worldwide. The speed of adoption and unprecedented nature of these measures left people unprepared, with many stranded abroad or separated from loved ones. It is thus during this period that social impacts were perhaps experienced most acutely. However, their prolonged use over several years, widespread introduction of other measure types (i.e., quarantine, testing, immunity certification), and frequent changes in how they were applied will have led to substantial additional impacts as individuals and populations sought to cope. The distribution of adverse social impacts are also likely to have been inequitably shared. There is need to better understand the social impacts of international travel measures throughout the duration of the pandemic.

Most (76%) of the reviewed studies used either qualitative or mixed methods to understand the lived experiences of impacted populations. The need to accommodate social distancing and geographical dispersion of study participants during the pandemic, however, affected how qualitative methods could be applied. Ethnographic and phenomenological approaches, which rely on detailed observation and engagement on site with study populations over time were particularly hindered. Community-based participatory methods were not used for these reasons. With the availability once again of in-person data collection, there are opportunities to gather deeper perspectives. For future research under pandemic conditions, innovations in the conduct of qualitative research using remote technologies should be more fully explored [29].

The travel measures analysed in the studies reviewed were not always stated explicitly, bundled together or with other non-pharmaceutical interventions, or inaccurately described. The term “border closures”, for example, was used to describe a varied range of restrictions limiting the entry and exit of travellers. Imprecise terminology can contribute to biased outcomes. For example, Liu et al. did not distinguish between COVID-related travel measures and immigration policy such as one-off residence temporary work visa in New Zealand [38]. Both are referred to as “border policies” and “border restrictions”. This undermines what studies identify as the independent variable causing social impacts. This review confirms the need for clearer articulation of the measures studied, separation of confounding measures, and standardized terminology and definitions to strengthen specificity and comparability of findings of social impacts [35].

Applying a standardized taxonomy [66] most studies focus on travel restrictions applied in varied ways, with two studies on quarantine [46, 56] and one study discussing testing and quarantine [7]. This may suggest that travel restrictions may cause the most social impacts although there remains a need to better understand the social impacts of other types of travel measures during COVID-19. For example, testing requirements were complex and often costly, raising concerns about access and affordability for some populations. Similarly, fulfilment of immunity certification requirements for travel required access to approved vaccines in a context of significant global inequity. This suggests any travel measure, if implemented without sufficient equity considerations, can cause adverse social impacts. In addition, most studies overlook the fact that various types of travel measures are often implemented together (e.g. testing and vaccination), that travel measures change over time, and their social impacts were shaped by how travel measures were used by other countries.

The reviewed studies cover the full range of categories of social impacts put forth by Vanclay [62]. The findings of almost all studies suggest multiple categories experienced simultaneously. For example, becoming stranded overseas due to travel restrictions can lead to employment loss and, in turn, precarity that impacts basic needs. However, the studies reviewed do not address this issue in detail. There is need to better understand the specific pathways by which international travel measures, especially when applied over a prolonged period, cause a chain of events leading to adverse social impacts. Further research on these causal pathways can inform policy decisions on how to effectively mitigate secondary outcomes by targeting impacts that are connected to others.

It is notable that, while most studies are understandably concerned with negative social impacts, there were also positive outcomes arising from the use of international travel measures. These range from the increased sense of solidarity, security and community as “silver linings” [9] to the achievement of an improved work-life balance by reduced international travel [22]. This suggests the need to better understand the distribution of positive and negative impacts within and across societies from the use of international travel measures. This will inform ways future decision making about such measures could seek to mitigate their negative impacts (e.g., use of exemptions, provision of special accommodations), and amplify the positive. This is especially important as public opposition to travel measures, and willingness to comply with public health orders, waned over time in many settings.

Importantly, improved understanding of the secondary outcomes from travel measures can inform policies to mitigate their effects on priority populations. For populations whose mobility is inherent to their livelihoods, such as digital nomads, cross-border commuters, transport workers such as flight crew and seafarers, and foreign migrant workers, priority may be given to mitigating their individual and population-level travel-related risks. Rather than blanket entry and exit restrictions, these populations may be provided with priority access to personal protective equipment, testing, vaccination and quarantine. Consideration may be given to their relative role in maintaining essential goods and services during a public health emergency, but also to the increased risks to their health and well-being. For populations who do not seek to travel internationally, but are adversely impacted by reduced volumes of travel (e.g., tourism sector, higher education sector), public funding to compensate for lost revenues may be considered. For populations reliant on social connectivity in other countries, such as diaspora, initiatives to support affordable virtual connection may be offered [54]. For example, the virtual observance of cultural or faith-based events and practices could be approved and expanded [47]. Advances in virtual reality was promoted as a substitute for holiday travel. Finally, increased consideration may be given to the special needs of refugees and asylum seekers. The closure of government and UN High Commissioner for Refugees offices hindered the ability to submit and be interviewed for a claim for asylum. Many countries stopped the processing of claims and restricted travel, resulted to substantial hardship and increased personal risk. Telephone or on-line (rather than in person) registration and interviews were permitted in a few countries (e.g. Germany). The expiration of visas, entry documents and other documentation, due to travel restrictions, should be waived. Procedures to enable the safe arrival of vulnerable populations during public health emergencies should also be developed including housing and access to healthcare. Unofficial migrants (without documentation) pose a particular challenge, given the unlikelihood of adhering to international travel measures such as screening and quarantine. An “ask no questions” policy, to enable access to PPE and other essential supplies, may be considered to enhance safe mobility regardless of the official status of the traveller.

### Limitations

There are two limitations to this scoping review. First, given variation in how the “social” realm is conceptualized and defined and described, there are likely impacts beyond the categories applied that may not have been captured. Second, given that many studies identified social impacts arising from a broad range of measures adopted in response to COVID-19, it was not possible to disentangle these effects specifically for international travel measures.

## Conclusion

The shift from WHO recommendations to refrain from the use of travel restrictions, during the early phase of the COVID-19 pandemic, to the application of risk-based approaches to applying different types of travel measures requires fuller understanding of their secondary outcomes. This scoping review confirms the significance of social impacts but there remain important knowledge gaps. Urgent attention to these knowledge gaps will support fuller understanding of how specific types of travel measures create positive and negative social impacts for diverse populations worldwide, and inform decision makers in the choices they make prioritising public health versus other policy goals during future public health emergencies.

## Data Availability

All data generated or analysed during this study are included in this published article.
